# Endo-Periodontal Lesions Without Root Damage: Recommendations for Clinical Management

**DOI:** 10.3390/jcm14186655

**Published:** 2025-09-22

**Authors:** Susana Aranda Verdú, Antonio Pallarés Sabater, Antonio Pallarés Serrano, Jorge Rubio Climent, Alberto Casino Alegre

**Affiliations:** 1 Department of Endodontics and Restorative Dentistry, Catholic University Valencia, 46001 Valencia, Spain; susana.aranda@ucv.es (S.A.V.); antonio.pallares@ucv.es (A.P.S.); antoniopallaresserrano@gmail.com (A.P.S.); jorrucli@hotmail.com (J.R.C.); 2 Doctoral School, Catholic University of Valencia, 46001 Valencia, Spain; 3 Oral Surgery Unit, University of Valencia, 46010 Valencia, Spain

**Keywords:** apical periodontitis, endo-periodontal lesions, periodontal, periodontal disease, regeneration, root canal treatment

## Abstract

**Background:** Endo-periodontal lesions (EPLs) are defined by a pathological connection between the pulp and the periodontium in a specific tooth. Although the identification of EPL etiology is of paramount importance for a correct treatment plan, it may be impossible at times to pinpoint the primary cause prospectively. A critical aspect of treatment planning involves assessing the necessity of root canal treatment in periodontal patients presenting with EPLs, evaluating the indication for periodontal intervention in cases of endodontic origin and determining whether splinting of the affected tooth is required. The aim of this article is to provide a concise perspective on the management of EPLs without root damage. **Methods:** A clinical decision-making flowchart was created based on current classification systems to guide treatment strategies for EPLs. **Results:** A flowchart of the treatment of EPLs based on possible decision pathways with a starting point in the diagnosis of the periodontitis or non-periodontitis patient that can prove particularly useful in cases where the initial etiology remains uncertain. **Conclusions:** EPLs are infections that represent a significant clinical challenge. This article summarizes key diagnostic considerations and therapeutic steps, offering practical guidance for clinicians managing these lesions.

## 1. Introduction

Periodontal and endodontic diseases are inflammatory responses to microbial contamination leading to periodontal and pulpal tissue damage and destruction [1,2]. Apical periodontitis caused by a pulp infection can cause periodontal involvement, just as an advanced periodontal lesion can cause pulp pathology [3,4]. In addition, periodontal and endodontic diseases can develop independently and simultaneously in the same tooth and coalesce as they progress [3].

An endo-periodontal lesion (EPL) is a pathological communication between the endodontic and periodontal tissues of a given tooth [2,5]. Microorganisms play a key role in EPLs. Studies show similar microbiota and inflammatory profiles in root canals and adjacent periodontal pockets. Some bacterial species are present in both sites, but with varying prevalence [6]. Clinically, EPLs are characterized by presenting an inflamed or necrotic pulp, with negative or altered response to pulp vitality tests, with deep periodontal pockets reaching or close to the apex and with periapical or furcation radiolucency [3,7]. Other signs and symptoms reported are pain, purulent exuded, tooth mobility, sinus tract and crown and gingival color alterations [2]. The identification of the etiology of EPLs is important for a correct treatment plan [8,9], although sometimes it is impossible to prospectively pinpoint the primary cause [3].

Traditionally, the etiological classification given by Simon et al. (1972) [10] has been used, and it distinguishes between five different EPL types from class I to V: primary endodontic lesions; primary endodontic lesions with secondary periodontal involvement; primary periodontal lesions; primary periodontal lesions with secondary endodontic involvement; and “true combined” lesions. Subsequently, it was simplified [11], and EPLs became classified as one of: an endodontic-periodontal lesion, a periodontal-endodontic lesion or as a combined lesion. Herrera et al. (2018) [2] proposed a broader classification whereby EPLs were divided into two groups: EPLs with root damage and EPLs without root damage. EPLs where root damage is ruled out are categorized as EPLs in periodontitis patients or EPLs in non-periodontitis patients. Both groups have been subclassified in terms of degree and according to the extent of periodontal destruction around the affected teeth: *grade I*, narrow deep periodontal pocket in one tooth surface; *grade II*, wide deep periodontal pocket in one tooth surface; and *grade III*, deep periodontal pockets in more than one tooth surface [2].

The new EPL classification introduced by Herrera et al. [2] has enabled us to develop a planning approach geared toward decision-making in the treatment of EPLs in the absence of root damage. With this proposal we do not need to know exactly from the beginning if the EPL etiology is either endodontic or periodontal.

The aim of this article is to offer a clinical update on EPLs of endodontic and/or periodontal etiology, through a proposed treatment protocol, thereby enabling clinicians to achieve higher rates of success, as well as long-term survival.

## 2. Methods

An electronic search of the PubMed and Medline databases was conducted to review and update the classification and management of EPLs. The search aimed to identify relevant articles and was carried out using keywords and synonyms (endodontic-periodontal lesions, endo-perio lesions, periodontitis, and periodontal surgery), with no restrictions on date of publication. The search also included a manual review of selected references and was limited to articles in English. Based on the most recent classification systems and current scientific publications, the authors developed a decision tree for the management of EPLs without root damage.

## 3. Results

### 3.1. EPL Diagnosis

The starting point is a tooth presenting a pathological communication between the pulpal and periodontal tissues, resulting in an acute or chronic condition that can be diagnosed as EPL.

The diagnostic examination of the affected tooth should include evaluation of tooth mobility, probing depth (PD), clinical attachment level (CAL), fistula tracking, pulp vitality test and radiographs [7]. It is mandatory in these pathologies to perform a small-field CBCT [12,13]. CBCT allows three-dimensional visualization of radiolucent lesions and the associated bone defects in EPLs, providing valuable diagnostic information regarding their location, size and extent. A high quality CBCT image can aid in identifying the cause of the radiolucent lesion, such as an omitted canal in a failed RCT or a lateral radiolucent lesion caused by a lateral canal. Additionally, CBCT provides critical information essential to conducting the differential diagnosis of EPLs with root damage, highlighting patterns of bone destruction associated to vertical root fracture, cracking, root canal or pulp chamber perforation or external root resorption [14]. The management of these situations is different from the protocols described in this article and its prognosis is more compromised [2].

In the diagnostic process it will be necessary, in addition to evaluating the problem tooth, to include the rest of the teeth to detect if we are treating a periodontal patient.

### 3.2. Management of EPLs Without Root Damage

**Figure 1:**
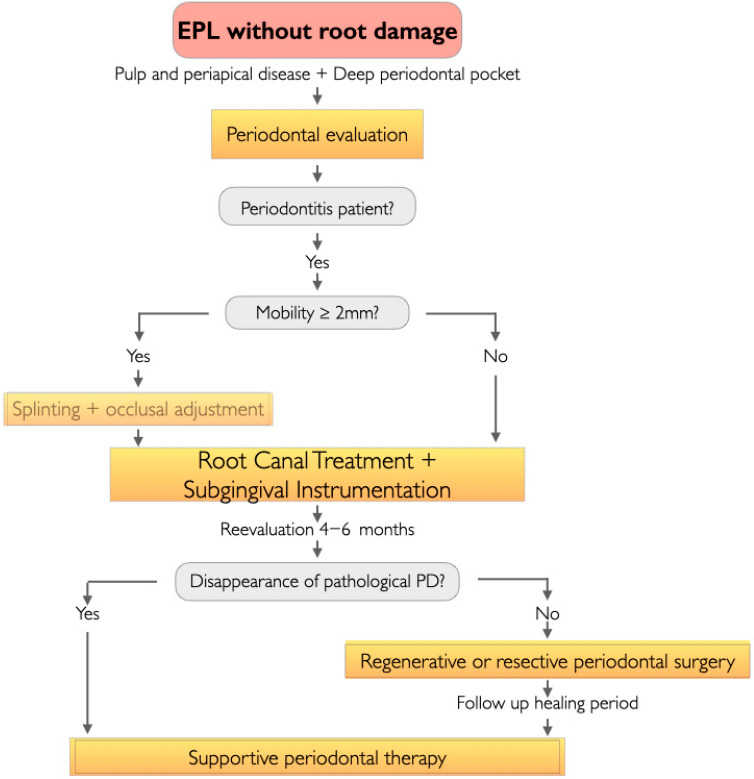
Clinical flowchart for the management of EPLs without root damage in the periodontitis patient.

**Figure 2:**
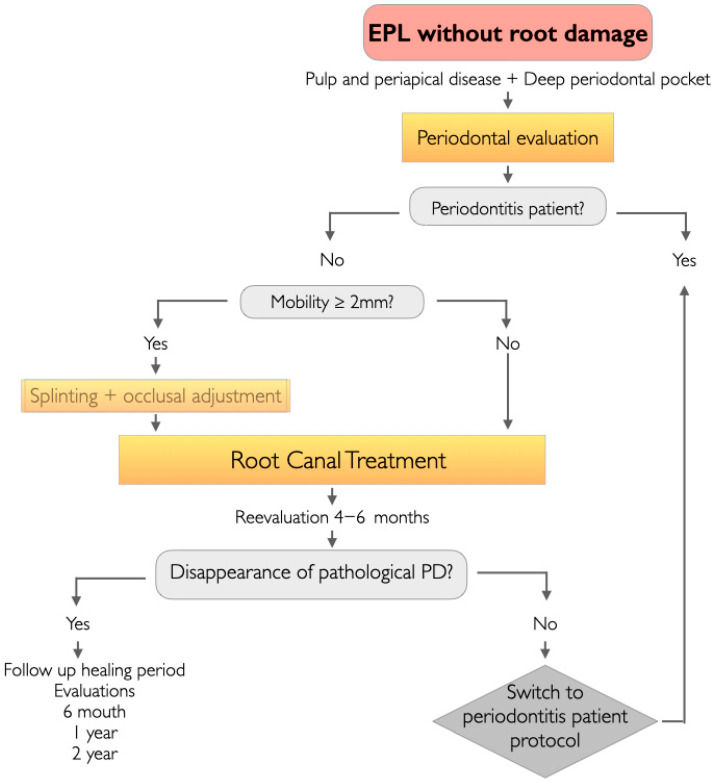
Clinical flowchart for the management of EPLs without root damage in the non-periodontitis patient.

For a tooth that does not present root damage and where we have diagnosed EPL, a decision-making flowchart for EPL management was formulated, depending on whether the patient presented periodontitis or non-periodontitis (Figure 1 and Figure 2).

The procedure involves an in-depth and comprehensive periodontal evaluation to determine the absence or presence of periodontitis in the patient. A patient is classified as a case of periodontitis when there is detectable interproximal CAL in ≥2 non-adjacent teeth, or buccal/lingual CAL of ≥3 mm with pockets of >3 mm detectable in ≥2 teeth. The observed CAL cannot be attributed to non-periodontal causes, such as dental caries in the cervical area, recession due to trauma or malpositioned teeth [5,15].

#### 3.2.1. EPLs in the Periodontitis Patient (Figure 1)

In the first step, we will assess the mobility of the tooth with EPL. Those that present mobility ≥ 2 mm should be splinted to the neighboring teeth and their occlusal adjustment be carried out by selective grinding [16,17]. Next, we will perform root canal treatment (RCT) or carry out retreatment and furthermore, periodontal treatment [16]. In diagnosed periodontal patients who are already in the treatment phase, a supportive periodontal therapy (SPT) session will be scheduled at the end of endodontic treatment, in which subgingival instrumentation of the tooth with an EPL will be repeated. In periodontal patients who have never received periodontal treatment, we will proceed with the diagnosis of periodontitis following the 2017 classification of periodontal and peri-implant diseases [15,18] and periodontal therapy consisting risk factor control, habit modification and subgingival instrumentation [19].

At 4–6 weeks we will perform a periodontal reassessment and schedule periodontal surgery in those sextants were necessary, except in the sextant of the tooth with an EPL if regenerative surgery is possible [16].

Three months after the start of non-surgical treatment, we conduct a clinical and radiographic evaluation of the tooth with EPL. Patients in whom the clinical symptoms of the EPL have been resolved, with their PD ≥ 4 mm and who do not require resective surgical treatment in that sextant for their periodontal disease will be transferred to SPT.

Otherwise, we will program periodontal regeneration surgery [20]. It will be necessary to determine the extent of periodontal destruction around the affected teeth and to plan the surgical approach. The choice of regenerative material is based on defect anatomy and the flap design chosen to expose the defect. Enamel Matrix Derivative (EDM) is ideal for containing defects and is the material of first choice; furthermore, minimally invasive surgical techniques are preferred [21]. If there are no containing defects and extended flaps are used, EDMs should be applied in combinations of barriers and fillers [21] or using ePTFE titanium reinforced membrane or bioabsorbable membrane with a filler material [22].

We then carry out follow up examination every week for the first month, every 3 months in the first year [16] and SPT every 3–6 months according to individual disease risk and susceptibility [19].

#### 3.2.2. EPLs in the Non-Periodontitis Patient (Figure 2)

Teeth with EPLs that present mobility equal to or greater than 2 mm should be splinted to neighboring teeth and perform occlusal adjustment by selective grinding, eliminating excessive occlusal force on this tooth [16,17].

We will exclusively perform RCT, or non-surgical endodontic retreatment, in cases where there is a failure of the initial RCT already performed.

The first reassessment visit takes place at 3 months to verify the absence of the symptoms and to check for the disappearance of any pathological PD caused by a sinus tract extended to the gingival sulcus or furcation area [4].

At 6 months an examination is recommended and annual follow up evaluations, then yearly for at least 2 years to confirm by X-ray the progressive healing of apical periodontitis [23].

In EPL cases of endodontic etiology in a non-periodontitis patient, RCT will achieve complete resolution of the case [3,4], without there being a need for any additional periodontal treatment. Only if pathological communication between the endodontic and periodontal tissues should persist over time would we conduct the treatment protocol described for periodontitis patients.

## 4. Discussion

A connection between endodontic and periodontal tissues is a requisite for the pathology to be considered an EPL. It is necessary therefore to make a differential diagnosis between EPLs and exclusive periodontal lesions, in which this communication does not exist. Simon’s classic classification includes a group of primary periodontal lesions without secondary endodontic involvement. For a long time, this has led to the mistake of considering that there could be EPLs that did not have pulpal involvement, and therefore, it was not necessary to perform RCT. Abbott and Salgado [24] pointed out the complications arising from Simon’s classification by considering “single-site” diseases (primary endodontic or primary periodontal conditions) and the difficulty in distinguishing between cases of primary lesions with secondary involvement and those of “true combined” lesions.

The effect of periodontal inflammation on the pulp has been controversial, and it is believed that it can only occur when periodontal disease generates a vertical bony defect that involves the apex [4]. CBCT verification of this type of defect will be key for the differential diagnosis between a EPL and an exclusive periodontal lesion.

Based on the EPL classification by Herrera et al. [2], we have designed a treatment decision flowchart. The starting point is to identify whether we are dealing with a periodontitis or non-periodontitis patient, instead of trying to discern whether the EPL has an endodontic or periodontal etiology or both. The difficulties in initially diagnosing the etiology of EPLs are due to the fact that the pulp vitality test is taken as a reference. Positive pulp vitality measurements have been associated with EPLs of periodontal origin and negative with EPLs of endodontic or combined origin. The pulp vitality test is an imprecise and low-reliability diagnostic test due to the possibility of false negatives or positives. Multi-rooted or single-rooted teeth with two or more canals may present partial necrosis in EPLs of pulpal etiology and be associated with a positive vitality test [7].

The etiology in EPLs of endodontic origin is related to bacteria from pulpal contamination, due to caries, trauma or incorrect previous RCT. These teeth present pulpal necrosis and apical periodontitis; therefore, endodontic treatment is essential. In the initial phases of EPLs of periodontal origin, we can obtain positive responses to the vitality test, in the face of a chronic inflammatory pulp, in which there is already contamination but there has not been time for total necrosis of the pulp to occur. Although some authors have suggested that RCT should only be performed in cases of negative vitality in EPLs of periodontal origin, it is recommended that the diagnosis be completed with the rest of the tests [7] and guide the treatment plan according to the proposed decision tree, depending on whether the patient is a non-periodontal or periodontal one and always performing RCT. Ruetters et al. [25] have pointed out that RCT is a highly predictable therapy for teeth in all cases of EPL. Cortellini et al. [20] recommend RCT for teeth with vital or necrotic pulp whenever instrumentation of the root apex was expected during the regenerative surgery, in the treatment of teeth with a seriously compromised prognosis, both in exclusive periodontal lesions and in EPL.

Consequently, RCT has been proposed in our protocol as an essential tool geared at treatment success in all cases of EPLs, regardless of their etiology.

Another classically raised question is whether periodontal treatment is necessary in EPLs of endodontic origin. Some authors have recommended performing RCT and subgingival instrumentation simultaneously in the initial phase of infection control in the treatment of EPLs [26,27]. However, in the case of EPLs in non-periodontitis patients, the only endodontic treatment in the case of a periradicular lesion due to a pulpal necrosis usually heals with adequate instrumentation, disinfection and sealing of the root canal system [1,8]. Non-surgical endodontic treatment is a predictable procedure, with a success rate in terms of prognosis in 83–97% of cases [28,29]. In these cases, it is recommended not to remove the cementum in order to create a favorable environment for periodontal regeneration [24].

Narrow and deep probing in EPLs of pulp-periapical origin is due to the presence of the sinus tract extending into the gingival sulcus or furcation area and may be mistaken for a periodontal pocket. This clinical situation is known as retrograde periodontitis, in which the sinus tract pathway traverses the periodontal ligament. This sinus tract disappears at an early stage once the RCT has been carried out [4].

We recommend a period of observation after undergoing RCT, because although the decrease in PD may occur in the first weeks, more time will be necessary for bone regeneration to be detected radiographically [8,9]. This period of time will allow the initiation of tissue healing, reduce the potential risk of bacterial contamination during the initial healing phase and prevent aggressive removal of the periodontal ligament and cementum, which could negatively affect periodontal healing [4]. Treatment results should be evaluated in 3 months and only should periodontal treatment be considered.

Tooth mobility has been associated with greater PD, clinical attachment loss and alveolar bone loss [17]. It has been shown that in teeth with increased mobility, there is not the same favorable response as in non-mobile teeth in terms of the clinical outcomes of regenerative surgery [21,30]. Occlusal therapy is usually accepted as a part of periodontal therapy. Splinting and occlusal adjustment are indicated in this protocol to manage EPLs with mobility ≥ 2 mm. The benefits of reduced EPL tooth mobility are to enable RCT in better conditions, increase the patient’s chewing comfort and favor the recovery of the periodontal attachment apparatus. Splinting also improves the prognosis of regenerative surgery if the tooth requires it.

EPLs in periodontal patients is associated with more compromised prognoses. Although in all EPLs the correct procedures and techniques will be key factors for the success of the treatment, the prognosis of EPLs in periodontitis patients will be influenced by other factors, such as the extent of the periodontal destruction, the need for surgical treatment, patient compliance with SPT, appropriate habit control and the patient’s general condition [19,21].

Wherever possible, surgery in relation to EPLs in the periodontitis patient should have a regenerative approach. Regenerative procedures have been widely used to restore lost periodontal tissue [19] and have been included to improve the EPL prognosis [16]. However, the success of endodontic microsurgery is significantly greater in isolated endodontic lesions than in EPLs [31,32]. Cortellini and Stalpers [20] reported cases where tooth retention was prolonged, generating an 88% survival rate at 10 years on teeth presenting bone loss at or beyond the root apex. Rosen et al. [33] point out that although regenerative periodontal surgery using GTR is the recommended option in many studies on EPLs, it is difficult to determine the benefit due to the lack of comparative studies with or without GTR in EPLs in the existing literature. New comparative studies could contribute to expanding this decision-making tree by providing specific recommendations on materials and techniques tailored to each clinical situation amenable to regeneration.

In patients in whom regeneration is contraindicated, as in such high-risk groups as smokers or subjects with poor plaque control, the adjunctive benefit of periodontal regeneration may be limited. Access or resective periodontal surgery may be an alternative to consider [21], as it will enable a deep pocket reduction, a clinical attachment gain [19], and thereby avoid disease recurrence, in spite of the undesired increase in gingival recession.

## 5. Conclusions

This article proposes a new approach in the clinical management of EPLs without root damage, through a decision tree based on whether we are dealing with a periodontitis or non-periodontitis patient. The diagnostic criteria to determine if a patient has periodontitis are clearly established and are easily objectifiable. In this way we prevent the weight of clinical decisions from falling exclusively on pulp vitality, which is a more imprecise diagnostic test. This proposal may serve as a useful tool for initiating the treatment of a tooth presenting an EPL when the causal diagnosis is initially unclear, a clinical scenario that is commonly encountered. RCT and mobility control through splinting are essential in the management of EPLs in periodontitis and non-periodontitis patients. In addition, subgingival instrumentation, periodontal surgery and SPT will have a fundamental role in periodontitis patients. Although more compromised prognoses have been attributed to EPL, correct planning and execution of cases can improve prognosis and lengthen tooth retention time, even in cases which had been considered as hopeless. There is a clear need for randomized controlled trials and long-term comparative studies to determine whether assessing the patient’s periodontal status at the initial diagnostic stage in the management of EPLs increases the likelihood of treatment success. Such research will be essential to strengthen the clinical evidence supporting the proposed recommendations.

## Data Availability

The data that support this study are available from the Catholic University of Valencia. Restrictions apply to the availability of these data, which were used under license for this study.

## Abbreviations

The following abbreviations are used in this manuscript:

EPL	Endo-periodontal lesion
PD	Probing depth
CAL	Clinical attachment level
SPT	supportive periodontal therapy
